# Dual feed progressive cavity pump extrusion system for functionally graded direct ink write 3D printing

**DOI:** 10.1016/j.ohx.2024.e00515

**Published:** 2024-02-12

**Authors:** Max J. Sevcik, Jacob Golson, Gabriel Bjerke, Isaac Snyder, Gage Taylor, Finnegan Wilson, Grace I. Rabinowitz, Dylan J. Kline, Michael D. Grapes, Kyle T. Sullivan, Jonathan L. Belof, Veronica Eliasson

**Affiliations:** a Colorado School of Mines Department of Metallurgical and Materials Engineering, Mechanical Engineering, Colorado School of Mines, Golden, CO 80401, United States of America; b Lawrence Livermore National Laboratory, Livermore, CA 94550, United States of America

**Keywords:** Additive manufacturing, Direct ink write, Dual feed extrusion, Progressive cavity pump, Functionally graded, G-code processing, Arduino

## Abstract

Material extrusion Additive Manufacturing (AM), is one of the most widely practiced methods of AM. Fused Filament Fabrication (FFF) is what most associate with AM, as it is relatively inexpensive, and highly accessible, involving feeding plastic filament into a hot-end that melts and extrudes from a nozzle as the toolhead moves along the toolpath. Direct Ink Write (DIW) 3D printing falls into this same category of AM, however is primarily practiced in laboratory settings to construct novel parts from flowable feedstock materials. DIW printers are relatively expensive and often depend on custom software to print a part, limiting user-specificity. There have been recent advancements in multi-material and functionally graded DIW, but the systems are highly custom and the methods used to achieve multi-material prints are openly available to the public. The following article outlines the construction and operation method of a DIW system that is capable of printing that can produce compositionally-graded components using a dual feed progressive cavity pump extruder equipped with a dynamic mixer. The extruder and its capabilities to vary material composition while printing are demonstrated using a Prusa i3 MK3S+ desktop fused filament fabrication printer as the gantry system. This provides users ease of operation, and the capability of further tailoring to specific needs.

## Graphical abstract

**Figure:**
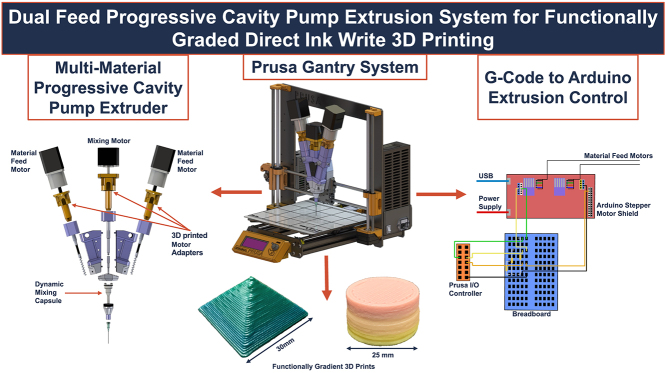

**Table:** 

**Specifications table**	

**Hardware name**	*Dynamic DIW Mixing Extruder for DIW 3D Printing (DDYNAMEX 3D)*

**Subject area**	Engineering and Materials Science

**Hardware type**	Mechanical Engineering and Materials Science

**Closest commercial analogue**	*Prusa i3 MK3S＋ MMU*

**Open source license**	TAPR OHL

**Cost of hardware**	*$21,500*

**Source file repository**	https://doi.org/10.17632/8953pcgdys.2

## 1 Hardware in context

Material extrusion additive manufacturing (AM) is the most widely practiced subcategory of AM recognized by ASTM standards, as it includes Fused Filament Fabrication (FFF). This means of AM is highly accessible, easy to use, and relatively inexpensive. FFF printing utilizes low melting temperature thermoplastic filament that is fed through a hot end nozzle, and quickly solidified in place of the toolpath. A related specific extrusion method that is commonly utilized in laboratory settings is Direct Ink Write (DIW) 3D printing. DIW is one of the most versatile methods of AM, as materials can be printed at ambient temperatures, as the only requirement for the material is for it to be able to flow out of an extrusion tool and hold form along a toolpath. The simple criteria of DIW makes it useful for printing a wide variety of materials including viscous polymers [1], [2], foods [3], [4], [5], solids-loaded slurries [6], [7], and reactive materials [8], [9], [10].

Many existing DIW systems used today are designed around specific process parameters that cater towards printing individual materials with a specific range of rheological properties. Toolheads used in DIW are typically designed to print parts composed of a single material. Designing a versatile DIW setup that permits multiple materials of a wide variety of rheological properties is highly sought after. The ultimate goal is to design a material independent DIW printer where processing parameters such as volumetric flow rate, retraction, and nozzle priming simply need to be adjusted when printing with new ink compositions [11].

Conventional 3D printers are typically designed to print a part with a homogeneous composition. Mounting more than one extrusion toolhead onto a printer unlocks the capability of multi-material DIW, increases the design space for complex heterogeneous components. As an example, Lawrence Livermore National Laboratory has demonstrated precise material deposition of multiple compositions with custom-built printers [12], [13]. Several systems capable of multi-material DIW have been created and demonstrate high resolution prints, however many of these systems are only capable of producing parts with a finite number of compositions, one for each tool head.

To further increase the design space capabilities for multi-material AM components, compositional gradients could be introduced to components to tailor properties spatially in a part. However, the capability to mix two feedstock materials in variable ratios as they are extruded adds another level of complexity. As an example, feedstock materials with short working times may need to be mixed shortly before deposition, realizing compositonal gradients requires specific consideration for volumetric flow rates, mixing residence times, and predicted spatial positions throughout a part. Active mixing and its efficacy of mixing has only been explored in the laboratory setting [14], [15].

Commercial DIW systems often utilize linear actuators to control extrusion, involving a screw driven plunger to push material out of a syringe. Although this method is simple and can be effective, it often limits users on the size of part that can be achieved. Parts cannot be larger than the ink cartridge that is intended to be used on the printer. Additionally, linear actuator printers can limit achievable geometric complexity, as stopping extrusion often results in nozzle leaks, especially when printing with low viscosity materials. When it comes to maximizing print fidelity and achieving discontinuous toolpathing, a progressive cavity pump extrusion system provides reliable and consistent results allowing for extrusion to start and stop seamlessly. Even with materials with lower viscosity that may be prone to leaking when extrusion has paused, a retraction and priming command scheme can be integrated used to mitigate leaking and to keep material at the tip of the extruder [11]. Progressive cavity pump dispensers are not limited by a single cartridge size, as long as material is constantly fed into the dispenser. The preeflow lineup of progressive cavity pump systems such as the ecoPEN series of are capable of accurately dosing silicone thermoplastics [16], ceramic slurries [17], and UV curing polymers [18], proving that extrusion, and therefore DIW 3D printing via progressive cavity pump is versatile and effective.

There are DIW systems available commercially that offer linear actuator DIW systems, capable of multi-material mixing extrusion. However, the hardware that is offered by these companies require the user to use their custom software to slice and print parts, and can cost up to $60,000 for the full scope of capabilities. Closed source DIW systems may be desirable for some, however may be limiting for those printing more exotic and challenging materials, where customization and the possibility of replication via open source guidance does not currently exist. The following article details a completely open source dual feed, single output mixing extruder, which the user can further modify to suit their DIW printing needs.

Mixing extrusion has been achieved in the past, utilizing static mixing nozzles. These nozzles work by receiving two feed materials that converge to an elongated nozzle tip containing obstacles that cause the two materials to mix together as they travel through the nozzle. Static mixing nozzles and are commonly used for mixing two-part adhesives and epoxies, and have been integrated into AM processes [19]. The mixing efficacy of these nozzles are highly dependent on the size of the static mixing nozzle, which may limit the user’s build volume in the Z-direction due to their length. Furthermore, static mixing nozzles are typically designed to achieve a single mixing ratio of the two feed materials, limiting users looking to achieve functionally graded prints.

In this work, compositionally graded prints have been achieved using a specialized progressive cavity pump system from ViscoTec (preeflow eco-DUOMIX) that utilizes two progressive cavity pump feeds which are combined inside of a dynamic mixing capsule. The resulting composition extruded from the nozzle is a ratio of input materials dictated by the ratio of feed rates between the two pumps. This system simultaneously controls feed of two different materials using specialized G-code commands and allows for mixing ratios to be changed on-the-fly during a print.

## 2 Hardware description

**Fig. 1:**
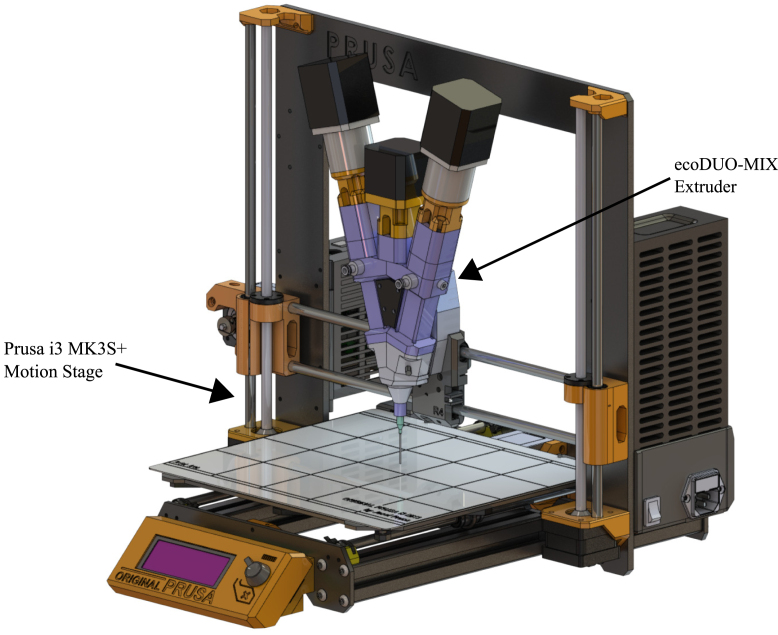
Render of modified preeflow eco-DUOMIX mounted onto Prusa i3 MK3S＋.

The following mixing extrusion DIW system consists of four main components. The gantry system **(1)**, which is the main frame of the apparatus, proving three-axis toolhead movement. The gantry is an independent machine which will interpret G-code analogous to a stand-alone printing system. The extruder **(2)**, which consists of a preeflow eco-DUOMIX series micro-dispenser from ViscoTec. The feed rotors have been modified to be driven via stepper motors instead of the original servo motors. These stepper motors are controlled by an Arduino stepper motor shield dosing control system **(3)** with G-code commands from the gantry control board. These G-code commands are implemented into the G-code through a post-processing script **(4)** after slicing that enables communication between the extrusion motors and the motion system. The following section will illustrate each component of the apparatus and describe how they work to make a fully functional DIW 3D printer capable of fabricating functionally gradient parts. Fig. 1 shows a rendered assembly of the full apparatus.

### 2.1 Gantry

The gantry system that provides toolhead movement for the preeflow eco-DUOMIX is a Prusa i3 MK3S＋ desktop 3D printer. This 3D printer was chosen as the gantry system due to its open source firmware. The original Prusa 8-bit Einsy RAMBo control board is used. Furthermore, the Prusa motion system provides highly repeatable and precise 3-axis movement due to its bipolar Nema-17 stepper motor controlled X, Y and Z axis, providing movement resolutions of 0.05 mm in each axis. The i3 MK3S＋ also comes standard with dual Z-axis, meaning two motors drive the Z-axis in tandem. This added power delivery to the axis means the printer can support the modified preeflow eco-DUOMIX, which has a mass of 1.5 kg.

### 2.2 Extruder and modifications

The eco-DUOMIX dosing system is generally paired with a dosing control box (preeflow eco-CONTROL EC200-DUO) allowing for the calibration of flow rates based on feed material density. This dosing control box has several settings that controls all three servo motors in tandem during extrusion at independent rates. Flow rates can be controlled by time, volume, or by the user through a electronic foot switch. These control systems are designed for simple, repetitive single-layer deposition of adhesives to electronics. For an assembly-line operation, this control system is effective in dosing consistent volumes, constant flow rates, and composition ratios. It is not designed for the dynamic extrusion rate changes required to accommodate a discontinuous 3D printer toolpath or material composition ratio changes during a print, and proved to be limiting in providing precisely timed dosing control, required for a multi-layered DIW setting. To achieve successful prints, dynamic extrusion control settings such as material retraction, nozzle priming, and dynamic flow rate adjustments are all needed, which the control box either lacked or could not have easily interfaced with the Prusa. Additionally, achieving material mixing ratio changes mid-print proved to be challenging to execute from the control box. For these reasons, it was decided that dosing control would not be operated through the eco-CONTROL EC200-DUO controller, and was not used in the final apparatus.

To achieve full control of the extrusion feed rate, stepper motors were used in place of the servo motors. The Marlin-based firmware used on the Prusa operates motors based on “steps” of rotation. Steps correspond to a known distance and correspond to rotation rate. To fit these stepper motors to the preeflow eco-DUOMIX, custom 3D printed parts were used to create a “key” fitting to allow the stepper motor axle to engage and rotate the material feed rotors. Due to the amount of torque required to rotate the material feed rotors inside of their stators, geared bipolar NEMA-17 stepper motors with a 5.18:1 gear ratio were implemented to ensure that extrusion would be possible, even with highly viscous materials. The middle mixing-capsule motor was replaced with the original Prusa extruder motor.

**Fig. 2:**
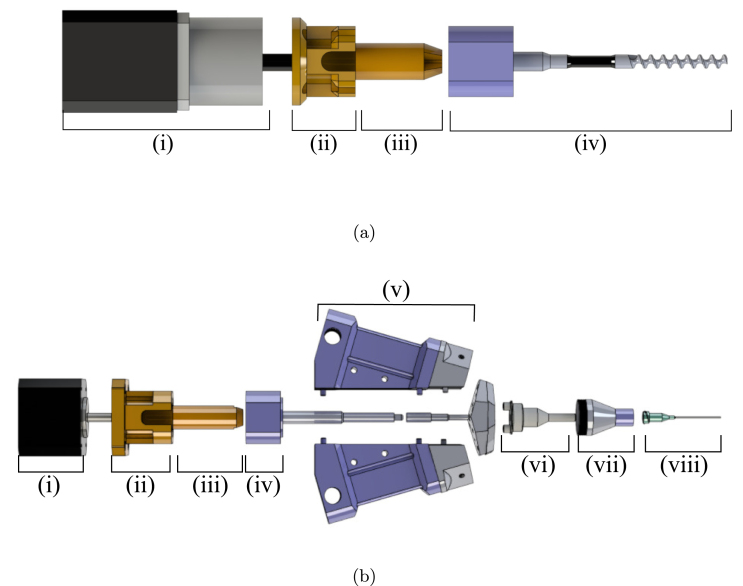
**(a)** (i) Geared stepper motor (ii) Geared stepper motor adapter (iii) Geared stepper motor shaft adapter (iv) Material feed auger. **(b)** (i) Stepper motor (ii) Stepper motor mount adapter (iii) Motor shaft adapter (iv) Mixing rotor (v) Material feed stator housing (vi) Dynamic mixing capsule (vii) Mixing capsule housing (viii) Extruder nozzle.

Fig. 2a illustrates an exploded View of custom 3D printed parts used to allow two geared stepper motors to drive the two material feed rotors. Fig. 2b is an exploded view of the of mixing component of extruder, showing how custom 3D printed parts interact with the mixing rotor, and how this induces mixing in the dynamic mixing capsule. The orange components denoted as components ii and iii in the figure indicate the custom parts that were designed and 3D printed to adapt the stepper motors to the ecoDUO-MIX dispenser.

### 2.3 Dosing control system

The eco-CONTROL EC200-DUO control box was replaced with an Arduino-Uno outfitted with a “CNC shield” to allow two additional stepper motors to be run simultaneously. This Arduino ultimately allows for live changes to the mixing ratio during a print. Once the materials for mixing have been chosen, a total extrusion rate is decided on and programmed into the Arduino. This rate correlates the extruder steps to the volume of material dispensed, for calibration purposes. The mixing system works by maintaining a constant sum of extrusion steps between the two material feeds. As the speed of feed A is decreased, the speed of feed B is increased such that the ratio of feed rate to total feed rate dictates mixing ratio while the volume extruded stays constant. The Arduino CNC Shield must be powered through an external DC power supply due to power requirements of the stepper motors.

The Arduino system works by utilizing data from the J19 header on the 8-bit Prusa Einsy RAMBo control board in conjunction with the open source “Accelstepper” stepper motor library. For this application, ports 6, 7, 8 and 14 (ground) on the Einsy RAMBo board were used to send G-code signals from the Prusa to the Arduino (Fig. 5). Digital signals on the J19 header pins can be activated using Marlin specific G-Code commands which set the pins to either a high or low signal. These high/low signals are sent to the Arduino stepper motor shield where an Arduino program receives them as a stream of digital signals. The signals are concatenated into a combination of eight bits, called bytes. Each unique byte representings a mixing ratio and extrusion direction based on the bit combination. Table 1 shows mixing ratios and their corresponding bytes in the Arduino program. This method allows for easy addition of mixing ratios or other commands to the Arduino script, as the ratio only needs to be added to this byte encoding scheme.

The bits are sent in a series of G-code commands. The first element in each line of a command, M42, addresses the J19 header. The second element, P[19/73/18], address different pins on the header. The third element, S[255/0], addresses each pin as either high, or low (digital 1 or 0). To write a full 8 bits to a stepper ratio control byte, 12 of these instructions are needed as a “block” of G-code commands. An example of this block is displayed below in Table 2.

**Table 1:** Bit combinations for each mixing ratio and direction.

Mixing ratio	Stop	5:1	4:1	3:1	2:1	1:1
bit 0	0	1	0	0	0	0
bit 1	0	0	1	0	0	0
bit 2	0	0	0	1	0	0
bit 3	0	0	0	0	1	0
bit 4	0	0	0	0	0	1
bit 5	0	0	0	0	0	0
bit 6	0	0	0	0	0	0
bit 7 (Direction)	0	0/1	0/1	0/1	0/1	0/1

The digital pins are always addressed in order of 19, 73, 18 in the G-code. Digital pins 19 and 73 are used as addressable bits to set the mixing ratios. Addressing these two pins in different combinations of either a high signal (255) or a low signal (0), called bits. Digital pin 18 acts as a bit translation, or “bit-check” signal to confirm the bits sent to pins 19 and 73, which are then sent to and read by the Arduino. The first time pins 19 and 73 are checked, pin 18 will be set to high (255), and the bits will be sent to the Arduino. Pins 19 and 73 will then be addressed again, and pin 18 will check pins 19 and 73 again, but this time sending a low signal (0). This process is necessary, as the execution of each G-code line occurs at an unpredictable time, thus sending these high and low bit-checks to allows the bits to be read in order. This cycle of checking high and low bits happens twice, leaving the user where each of the 8 bits can be addressed as high or low, giving the user 256 possible combinations for mixing ratios. The Arduino code provided in the file repository dedicates the final bit (bit 7) to change the direction of rotation of the stepper motors, allowing for nozzle retraction to prevent leaking after extrusion is paused. Therefore, bits 0 through 6 encode a specific mixing ratio. This allows for 128 possible non-integer ratios to run the stepper motors during extrusion. It should be noted that the same process can be carried out with only 4 bits, however this dramatically reduces the amount of possible mixing ratios that can be called on at a time, especially if the user desires to designate a bit to retract material when pausing extrusion, or the ability to reverse each mixing ratio. Since the preeflow ecoDUO-MIX mixing capsules claim designed to extrude at ratios between 1:1 and 5:1, 8 bits gives more than enough options to create all practical non-integer ratios between those whole ratios if needed.

**Table 2:** 4:1 Ratio+ (Extruding) G-code command block Example.

Command	Pin	High/Low	Function
M42	P19	S255	Bit 0
M42	P73	S0	Bit 1
M42	P18	S255	Check Pins High
M42	P19	S0	Bit 2
M42	P73	S0	Bit 3
M42	P18	S0	Check Pins Low
M42	P19	S0	Bit 4
M42	P73	S0	Bit 5
M42	P18	S255	Check Pins High
M42	P19	S0	Bit 6
M42	P73	S0	Bit 7 (Motor Direction)
M42	P18	S0	Check Pins Low

### 2.4 G-code post processor

A post-processor is used to input the Arduino communication G-code into the G-code extracted from the slicer software. A simple find and replace MATLAB script was created that searches through the desired G-code file, and replaces lines where extrusion is altered with the desired Arduino G-code blocks in order to start and stop extrusion. The post-processor calculates where to switch extrusion ratios based on user input for composition range and number of layers.

G1 EXXX // Begin Priming

G1 E-XXX // End extrusion after retracting x mm of filament, wait for begin extrusion command.

### 2.5 Hardware overview summary

• This dual feed mixing extrusion system utilizes an already existing robust design of a progressive cavity pump dispenser with dynamic mixing capabilities.
• High resolution dispensing capabilities from the VistoTec ecoDUO-450 extruder allows for discontinuous extrusion, making it highly compatible for DIW 3D Printing.
• Using a simple to use, open source desktop printer from Prusa Research, modifications to the gantry system to make preeflow ecoDUO-450 the primary extruder is possible.
• This system takes advantage of 3D slicers and G-Code generators that are already fully realized and optimized for precise toolpathing.
• Post-processing is necessary, but made simple through a script that adds new extrusion commands to allow multiple extruder motors to be run at once, and at different mixing ratios.

## 3 Design files

Table 3 encapsulates downloadable files that are used to construct and operate the following hardware. All files are provided with the article. The STL files provided are intended to be 3D printed to adapt stepper motors to the ecoDUO-MIX extruder. The Arduino files are used to drive the stepper motors that control extrusion. The MATLAB file is used to process new G-code files to include custom G-code to incorporate all three stepper motors at once while printing. Included is also the bill of materials, which includes all of the materials and supplies that are needed to operate the hardware.

**Table 3:** Files available with the article.

Design file name	File type	License	Location of the file
Geared Stepper Motor Mount	.STL	TAPR OHL	Available with the article
Geared Stepper Shaft Adapter	.STL	TAPR OHL	Available with the article
Mixing Stepper Motor Mount	.STL	TAPR OHL	Available with the article
Mixing Stepper Motor Shaft Adapter	.STL	TAPR OHL	Available with the article
eco-DUOMIX X-Gantry Mount	.STL	TAPR OHL	Available with the article
PINDA Adapter	.STL	TAPR OHL	Available with the article
Prusa-DUOMIX Communication Tester	.ino	TAPR OHL	Available with the article
Prusa-DUOMIX 4-bit G-Code Communicator	.ino	TAPR OHL	Available with the article
Prusa-DUOMIX 8-Bit G-Code Communicator	.ino	TAPR OHL	Available with the article
Prusa-DUOMIX G-Code Post-Processor	.m (MATLAB)	TAPR OHL	Available with the article
Bill of Materials	.xlsx	TAPR OHL	Available with the article

### 3.1 Brief description of files

• **Geared Stepper Motor Mount:** Plastic 3D printed part used to mount the geared stepper motors to the extruder rotors. Secured using four 40 mm M3 screws to secure to the extruder, and four 8 mm M3 screws to secure to the stepper motor. Two of these are required, as each extruder stepper motor will utilize this piece. See Fig. 2a, component ii.
• **Geared Stepper Motor Shaft Adapter:** Plastic 3D printed part that fits on geared stepper motor axle to allow compatibility with extruder rotor. Two of these are required, as each extruder stepper motor will utilize this piece. See Fig. 2a, component iii.
• **Mixing Stepper Motor Mount:** Plastic 3D printed part used to mount the middle mixing motor to the preeflow eco-DUOMIX. Secured using four 25 mm M3 screws to secure to the extruder, and four 10 mm M3 screws to secure to the stepper motor. See Fig. 2b, component ii.
• **Mixing Stepper Motor Shaft Adapter:** Plastic 3D printed part that fits on middle mixing stepper motor axle to allow compatibility with mixing rotor. See Fig. 2b component iii.
• **eco-DUOMIX X-Gantry Mount:** Plastic 3D printed part used to mount preeflow eco-DUOMIX to the X-Axis where the original extruder was. This is a friction fit, but has the option to be further secured using 40 mm M3 screws and hex nuts as well. This component is shown in Fig. 4b.
• **PINDA Adapter:** Attachment for the PINDA magnetic bed leveling sensor that allows for a simple friction fit onto the eco-DUOMIX and can be removed during printing. This component is shown in Fig. 7.
• **Prusa-eco-DUOMIX Communication Tester:** Arduino script that allows the user to test the communication between the Arduino and the two stepper motors. Instructions in the file walk the user through operations.
• **Prusa-eco-DUOMIX 4 Bit G-Code Communicator:** Arduino script that will allow the user to use G-Code commands to address different extrusion ratios, and alter the extrusion relative extrusion speed. The 4 bit version was originally used while developing the hardware and is a simplified version.
• **Prusa-eco-DUOMIX 8 Bit G-Code Communicator:** Arduino script that will allow the user to use G-Code commands to address different extrusion ratios, and alter the extrusion relative extrusion speed. The 8 bit version allows the user to address more mixing ratios, as well as reverse the motors on command. This should be used for more geometrically complicated prints.
• **Prusa-eco-DUOMIX G-Code Post-Processor:** MATLAB G-code processing script. This script will prompt the user to upload G-Code file, look for commands indicating the beginning and end of extrusion, and replaces the commands with the G-Code command blocks that control extrusion using the Arduino stepper motor shield. This post-processor simply makes extrusion possible with the user desired mixing ratios, however, this script can be added upon to initiate ratio changes during part manufacturing.

## 4 Bill of materials

A comprehensive list of all materials required to create and operate this hardware can be found in the file repository available with the article. In addition to the materials required for this hardware, various software will need to be installed as well, listed by Table 4.

**Table 4:** Software required to set up and operate the device.

Software	Link	Price
PrusaSlicer	https://www.prusa3d.com/page/prusaslicer_424/	Free
Pronterface	https://github.com/kliment/Printrun/releases/tag/printrun-2.0.1	Free
Arduino.IDE	https://www.Arduino.cc/en/software	Free
Visual Studio Code	https://code.visualstudio.com/download	Free
Simplify3D	https://www.simplify3d.com/	$199
Prusa Firmware	https://github.com/prusa3d/Prusa-Firmware	Free

## 5 Build instructions

### 5.1 Prusa printer assembly and testing

• Fully construct Prusa i3 Mk3S＋ using the included instructions. It is imperative that the entire apparatus is constructed as intended to ensure the system operates properly during printing, and that the product is not defective.
• It is highly recommended that the full setup wizard is conducted after the printer is constructed, as well as conduct some test prints using the stock filament. To test whether the printer is working, the several .STL files included in the file repository can be printed. These parts will need to be printed to mount the stepper motors onto the ecoDUO-MIX. The required modification components were constructed using PETG, however the stock PLA that comes with the Prusa i3 kit will suffice. The 3D printed parts experience minimal load during operation, however it is advised to print duplicates of each part.

### 5.2 Prusa filament extruder dissasembly

**Fig. 3:**
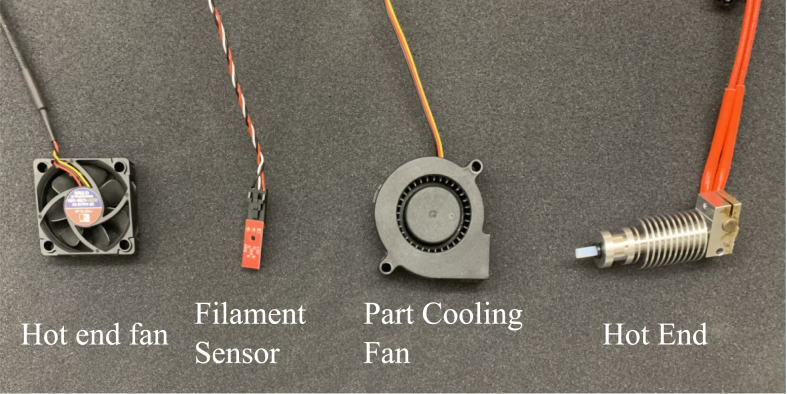
Remove these components from the apparatus and disconnect from the Prusa Einsy RAMBo board.

**Fig. 4:**
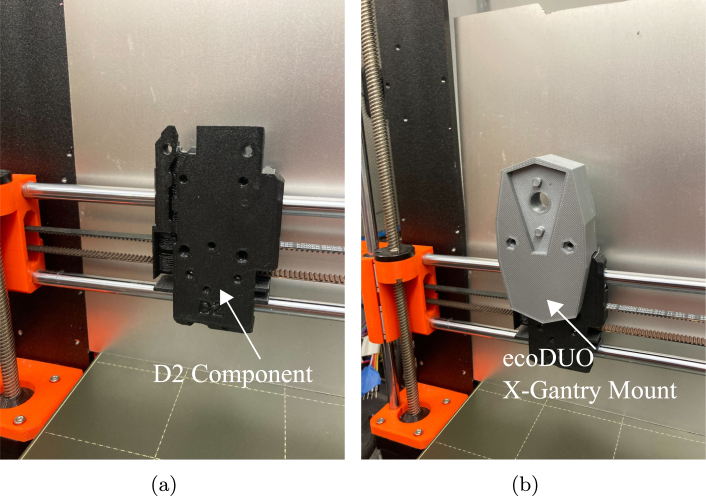
(a) Prusa X-Gantry with dismantled extruder mount. (b) X-Gantry with 3D printed eco-DUOMIX mount attached.

• Deconstruct stock Prusa i3 MK3S＋ extruder head from the X-axis of the gantry. Refer construction manual for the Prusa printer, and work backwards from the section that details the assembly of the extruder. Begin this process by disconnecting the hot end fan, filament sensor, part cooling fan, and hot end components from the Einsy RAMBo motherboard. Fig. 3 also shows the components that will be disconnected and removed.

• When deconstructing the extruder, leave the extruder stepper motor, thermistor, and magnetic bed leveling sensor (PINDA) attached to the Einsy RAMBo motherboard. The extruder stepper motor will be used as the driver for the mixing rotor on the preeflow eco-DUOMIX, and the magnetic bed leveling sensor will be modified to be a removable component to ensure bed leveling before a print. The thermistor from the hot end of the extruder must remain connected to the motherboard, as safety features built into the Prusa firmware forbid operation if the thermistor is not connected.
• Once the extruder is deconstructed, only the X-carriage components that encase the bearings which aid movement across the X-Axis smooth rods This plastic part will be have “D2” debossed on it. older versions of the Prusa i3 Mk3S＋ models may be labeled as “R4”, however the component is equivalent. Fig. 4 shows what the X-Axis gantry should look like after the disassembly is complete.

### 5.3 Firmware flashing

• Update the printer with the most recent firmware available for the Prusa i3 MK3S＋ from Github. The link is provided in Table 4. Follow the instructions on the webpage to access the required files using Visual Studio Code. The firmware utilized when developing this hardware was version 3.13.1
• In the Configuration_PRUSA_H.h file in Visual Studio Code, uncomment the following lines:
  – #define DEBUG-DISABLE-FANCHECK
  – #define DEBUG-PREVENT EXTRUDER
  – #define DISABLE-MINTEMP
  Disabling these lines is required to be able to allow cold extrusion, and bypass safety features that prevent prints without cooling fans. Ensure that the new firmware files are saved and compiled
• To upload the updated firmware file, PrusaSlicer can be used. Under the configuration tab, choose flash firmware, find firmware.ino.prusa-einsy_rambo.hex

### 5.4 Arduino setup

**Fig. 5:**
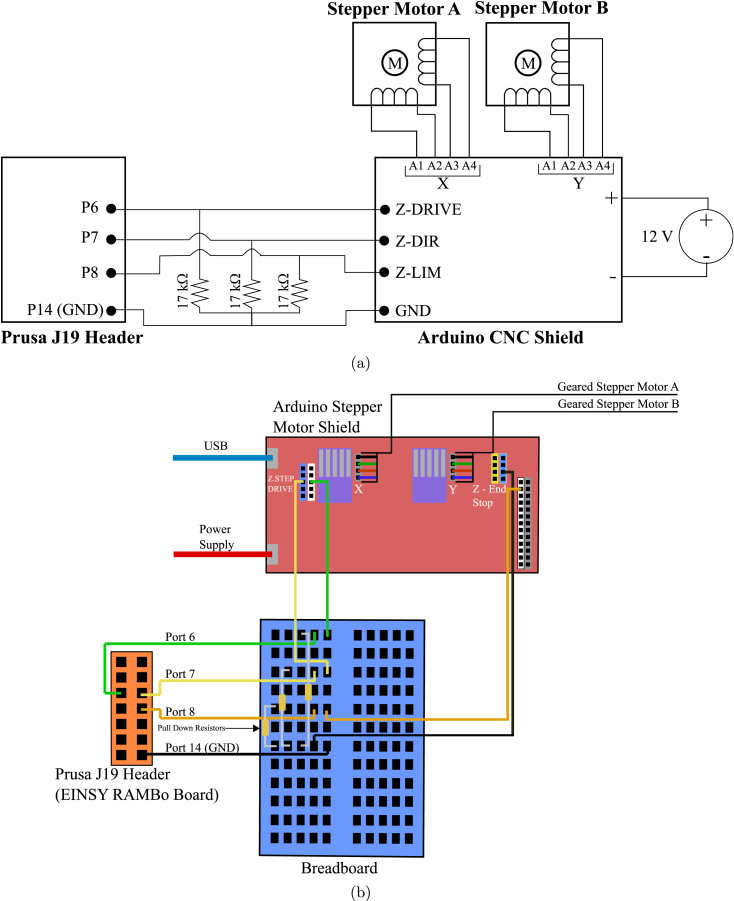
(a) Circuit diagram connecting the Prusa J19 header on the Einsy RAMBo control board and Arduino stepper motor shield. The system uses three 17 kOhm pull down resistors to maintain the default logic level to low. (b) Graphical schematic to allow communication between the Prusa printer and the Arduino.

• Set up breadboard and Arduino according to Fig. 5. Attach connections from the breadboard to J19 header pin on the Einsy RAMBo board. Attach power and ground to 12 volt power supply.
• Download all of the Arduino files from the repository.
• Download and install the Accelstepper stepper motor control library to the Arduino environment. The link is provided in the header of the Arduino file scripts.
• Upload the Prusa-DUOMIX-Tester.ino to Stepper motor shield Arduino. Open the Arduino Serial Monitor, and input various mixing ratios (as integers) to the serial monitor to verify that the stepper motors and their wiring has been installed correctly. Ensure that the motors are rotating the same way, and are rotating counter-clockwise when the axle is facing downward when positive ratios are input into the serial monitor. To flip the default direction, reverse the pin order on the CNC shield.

### 5.5 ecoDUO-MIX extruder assembly

**Fig. 6:**
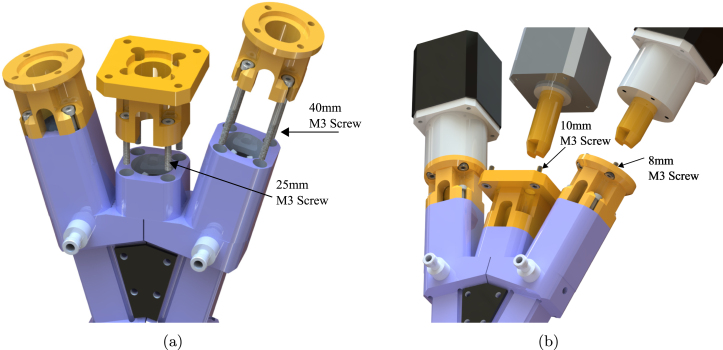
(a) 40 mm M3 screws are used to secure the geared stepper motor adapters to the ecoDUO-MIX. 25 mm M3 screws are used to secure the mixing stepper motor adapter. (b) 8 mm M3 screws are used to secure the geared stepper motor to the adapters, and 10 mm M3 screws are used to secure the mixing stepper motor to its adapter. The shaft adapters must be fit onto the axles of the stepper motors before assembling.

**Fig. 7:**
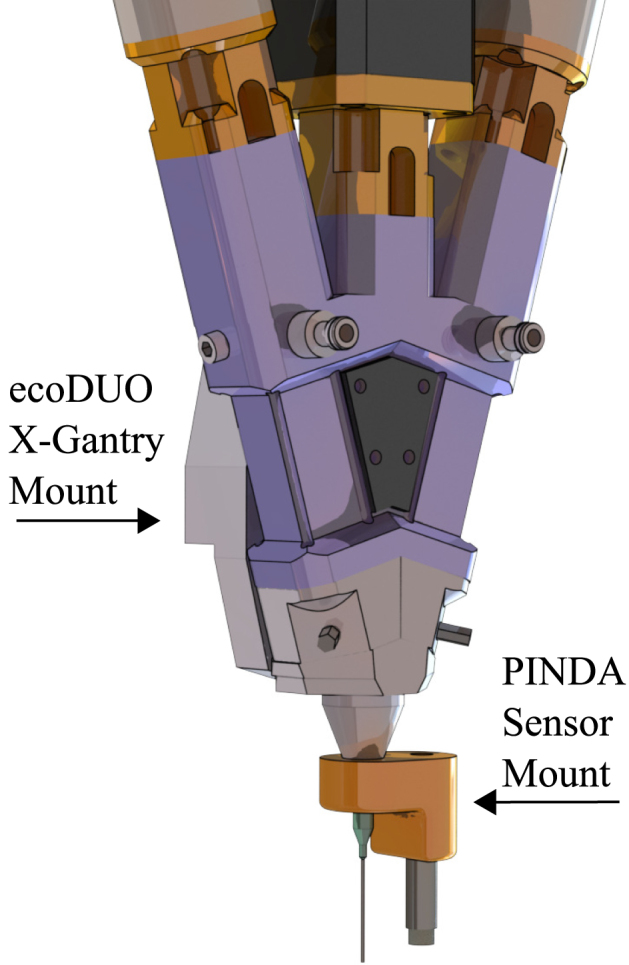
Customized ecoDUO-MIX with magnetic PINDA sensor attached. This piece is friction fit to the end of the nozzle, and is simple to attach and detach before conducting a print.

• Remove all three stock servo motors on preeflow ecoDUO-MIX 450. The material feed servo motors will be replaced with the with geared NEMA-17 stepper motors, and the mixing servo motor will be replaced with the Prusa extruder stepper motor.
• Using the 3D printed adapter parts, secure the stepper motor adapters to their respective locations indicated by Fig. 6a using 40 mm M3 screws. The mixing stepper motor adapter uses 25 mm M3 screws.
• Secure the 3D printed stepper motor shaft adapters to the shafts of the stepper motors according to Fig. 6b.
• Secure the geared stepper motors to ecoDUO-MIX geared stepper motor mounts using 8 mm M3 screws according to Fig. 6a.
• Secure the mixing stepper motor to the ecoDUO-MIX using 8 mm M3 screws according to Fig. 6b.
• Attach 3D printed PINDA Adapter part onto magnetic bed leveling PINDA. the wire attached to the sensor must be temporarily disconnected from the Einsy RAMBo board, and fed through the 3D printed part. This will be mounted onto the end of the preeflow eco-DUOMIX before printing to ensure proper bed leveling before printing ensues seen in Fig. 7. The end of the sensor should be 1.5 mm shorter than the nozzle when mounted onto the extruder.

### 5.6 Miscellaneous setup

• Assemble pressurized air assembly for ink cartridges. The pressure must be adjustable, and the outlet must be split to pressurize both ink cartridges at the same time.

## 6 Operation instructions

• Mix and load each ink component into their respective ink cartridges. Insert the plastic piston behind ink inside cartridge.
• Assemble preeflow ecoDUO-MIX with stepper motors using the 3D printed mounting components and mount the extruder onto the X gantry. The original extruder stepper motor will be attached to the mixer motor position on the eco-DUOMIX.
• Attach filled ink cartridges to the preeflow ecoDUO-MIX.
• Power on Prusa printer, as well as 12 volt power supply to stepper motors.
• Slice 3D part using slicer of choice with positioning in the middle of the print bed. The toolhead settings should be reminiscent of the nozzle used on the extruder. Printing speed should be reduced to around 400-600 mm/min to start. Table 5 displays benchmark tool settings used for 30 wt% cake icing formula, which was the material used in the following hardware validation section. Prime distance in Simplify3D is equal to the retraction distance if it is set to 0 [mm], therefore a non-zero value is additional priming relative to the retraction. Negative priming values will result in a priming distance that is less than the retraction distance. Ensure that the print bed temperature and the hot-end temperature set to 0 in the tool settings.
• Use the MATLAB post-processor script to add custom Arduino start/stop code blocks to G-code to allow for control over ink component A and B extruder motors
• Upload the post-processed G-code to Pronterface.
• Connect the 3D printer to Pronterface using a USB connection.
• Level print bead with toolhead using a G28 command. Ensure that the magnetic PINDA sensor is attached to the extruder, as it is what calibrates the Z-height. If the PINDA sensor is not attached to the extruder, it will crash into the bed.
• Set the absolute zero position in the Z-direction using a feeler gauge. Use a gauge leaf that is the same as the layer height. The nozzle should barely touch the feeler gauge, and this absolute position should be set to using “G92 ZX” command, where X represents the first layer height. This step is very important to ensure the first layer of the print is of high quality.
• Set E-Steps to 3500 [steps/mm] to set mixing motor speed. Done by sending the command “M92 E3500” to the printer using Pronterface, and overwriting previous settings by sending “M500”.
• Using Pronterface, jog the extruder to the middle of the bed, and raise the Z-height so it is high enough to remove the PINDA sensor. The PINDA sensor does not need to be attached to the extruder during printing.
• Prime the extruder cavities. This can be done by uploading the file “Prusa-DUOMIX Communication Tester.ino” to the Arduino. To communicate with the extruder open the Serial Monitor window by selecting Ctrl＋Shift＋M with the Arduino window open. Input the desired ratio into the prompt, and press ENTER. This ratio should be the same as the first ratio that is intended to be printed. Ensure that both material feed stepper motors are rotating counter-clockwise. Simultaneously, run the mixing stepper motor by selecting the “Extrude” button in Pronterface, below the jogging controls. It may take a few minutes for material to feed the cavity and deposit from the nozzle, especially when priming at uneven mixing rations. When the extruder is primed, input “0” into the Serial monitor to stop the motors.
• Upload the file “Prusa-DUOMIX 8-bit G-Code Communicator.ino” to the Arduino.
• Print part
• After printing, completely disassemble nozzle for cleaning, after depressurizing the material feed cartridges. Each component should be cleaned with a solvent that will effectively clean extruder components without damaging the extruder components.

**Table 5:** Parameters and the respective ranges chosen for DIW ink used in hardware validation.

Parameter	Value
Nozzle diameter	0.84 [mm]
Prime	0.72 [mm]
Coasting	0 [mm]
Toolpath speed	400–600 [mm/min]
Volumetric flow rate	200 [steps/min]
E-Steps	3500–5000 [steps/mm]

## 7 Maintenance

• Make sure that the ecoDUO-MIX dispenser is completely cleaned before each use. Failure to do this may result in blockage of the extruder.
• Allow all components of the extruder to completely dry before reassembling the ecoDUO-Mix dispenser. Pressurized air can be used to blow cleaning solvent or water from hard to reach areas of each part.
• Replace O-rings if lost or damaged. Leaking of feed material may occur if the O-rings are missing, or not properly installed.
• If cartridges have air blowing past the piston while pressurized, add vacuum grease to the outside edge of the piston, and inside walls of the cartridge. The vacuum grease will accumulate on the cartridge piston as it feeds material into the extruder.
• Ensure that the ends of the cartridge air supply adapters have been cleaned after each use. Material can build up around the interface of the O-rings, and can cause air leaks.
• The 3D printed components that adapt the stepper motors to the ecoDUO-MIX extruder may experience wear after some time. These parts, especially the shaft adapters may need to be replaced.
• Refer to the maintenance section of the manual of the ecoDUO-MIX dispenser system for further inquiries on extruder maintenance.
• For Prusa motion stage related maintenance, refer to the manuals included with the Prusa 3d printer kit.

## 8 Safety

The preeflow ecoDUO-MIX is not a food-safe tool approved by the US Food and Drug Administration. It is up to the user’s discretion whether or not food products printed by this hardware are safe to consume. If the user does desire to print food with this hardware, it is advised that only food products should ever be dispensed from the preeflow ecoDUO-MIX. The items in the following section were printed for demonstration and validation purposes.

## 9 Validation and characterization

Concept validation and test printing seen in the following figures below. All prints were achieved with a feed material consisting of a cake icing and granulated sugar mixture, where each of the two components were dyed a different color. The 30cc cartridges were prepared using pre-dyed cake icing [11]. Each cartridge was loaded with roughly 40 grams of icing and degassed using a FlackTek SpeedMixer DAC 330–100 Pro centrifuge mixer to eliminate air bubbles that can cause detrimental defects to a part during the printing process. Cake icing was chosen to evaluate the print qualities for this system as it proved to be self-supporting with consecutive layering, has no limit to working time, and proved to be easy to clean after printing. Additionally, choosing two feed materials with the same composition, but different colors showcases the possibilities to change mixing ratios during a print, and clearly demonstrates capabilities to achieve a functionally graded print.

Other materials have been successfully printed with this hardware, such as a thermoset PDMS with solids loaded calcium carbonate. The preeflow ecoDUO-MIX is capable of printing most materials with viscosities ranging from water to thick resins. However, solids loaded cake icing was chosen as the material to validate the hardware, as the color changes between two materials clearly indicates that the mixing ratio has successfully changed. UV curing materials can also be printed with this hardware, as additional components can be integrated to the system, such as a UV curing light.

**Fig. 8:**
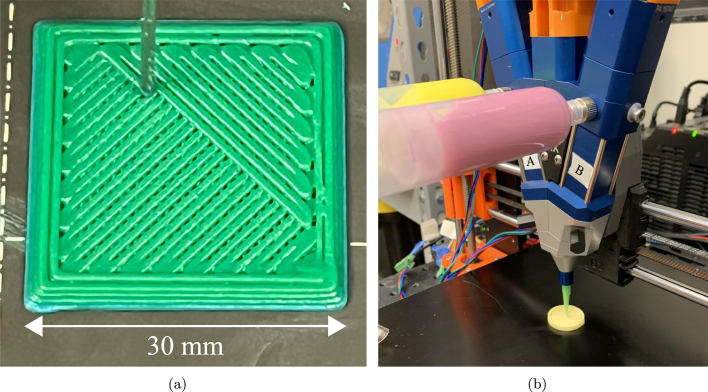
(a) In-situ image during a print with green cake icing illustrating the system’s precise toolpathing capabilities. (b) Image taken while printing a functionally graded cylinder. Labels for A and B indicate each feed. At this moment the A:B mixing ratio of the two feed materials is 4:1.

High infill density toolpathing is typically desirable for certain DIW printing operations, as printing voids in materials that are prone to flowing after deposition will not maintain their shape over time. Fig. 8 demonstrates the high resolution printing capabilities the system allows, as well as the capability for high infill densities.

**Fig. 9:**
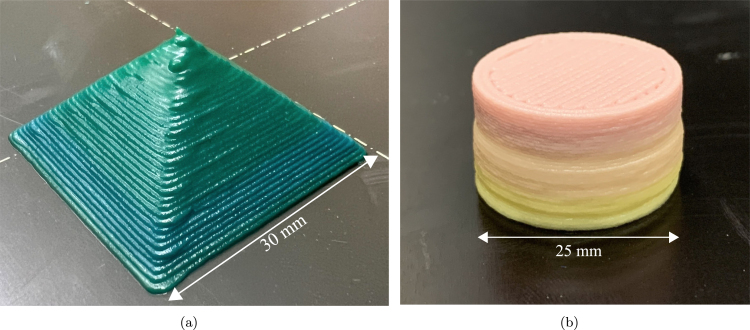
(a) Square pyramid with a 30 mm base. Blue–green ratio begins at 4:1 and ends at 1:1. (b) Cylindrical color gradient print showcasing a full spectrum of mixing ratios in a single print, beginning with a 1:4 mixing ratio, and ending at a 4:1 mixing ratio.

Since toolpath generation and execution is carried out on a gantry system that is capable of high resolution movements with highly adjustable acceleration parameters, the DIW system is capable of executing G-code similar to a FFF 3D printer. Part resolution is limited solely by the nozzle diameter on the extruder. Furthermore, geometries may be limited by feedstock material choice, as some highly viscous materials may be able to achieve higher aspect ratio prints without experiencing excessive slumping. Fig. 9 illustrates a print where the mixing ratio of the two feed materials was altered at different layers in the build direction, along with a demonstration of compositional variance in the build direction, showcasing the full spectrum of mixing ratios achievable on the system.

The advantage to creating a DIW system that operates on already existing desktop 3D printing firmware is the access to the settings for finely tuning print parameters available in commercial slicing software. This capability gives the user the freedom to print a wide range of materials with varying rheological properties, and simply adjust extrusion parameter settings when printing with a new material. For parameter selection, see Sevcik 2023 [11].

**Fig. 10:**
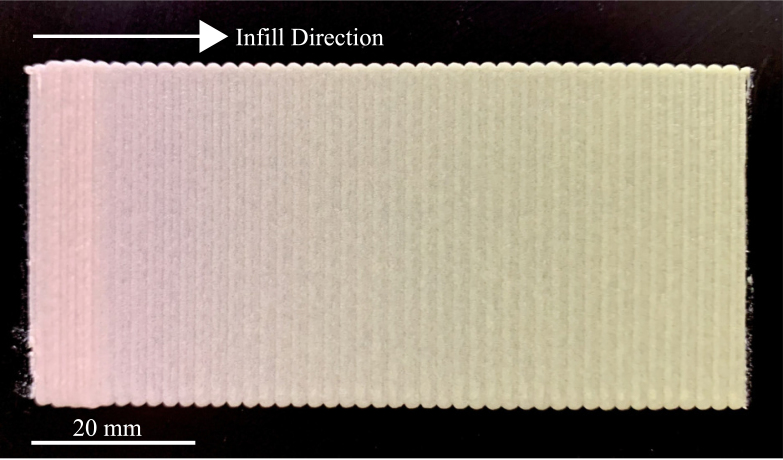
Mixing ratio change overlap during printing. The mixing ratio changes from 1:4 to 4:1 when the infill of the first layer starts (arrow indicates infill direction). The first indication of color change occurs roughly 8 mm into the infill direction, and completes 50 mm along the infill direction.

The buffer between feed ratios is an unavoidable factor of the system, as the dynamic mixing capsule must be dispensed before the new desired ratio is achieved. The volume of the dynamic mixing capsule, roughly 1 mL of material must then be dispensed before the feed ratio changes. Fig. 10 simulates a ratio change occurring during a print. Before this print was conducted, the feed ratio was a yellow-red ratio of 4:1, and was changed to 1:4. It can be seen that a noticeable color change begins 10 mm through the infill pattern, and a gradient to the new feed ratio lasts through roughly 50 mm. If the user desires a hard change between mixing ratios, a purging step can be incorporated into the G-code post processing script, where the toolpathing will pause, the nozzle will move to an unused region of the print bed, and extrusion will continue until the new feed ratio is achieved, in which the print can resume. The current G-code post processing script does not incorporate this feature, but can be implemented by the user if this is desired.

The hardware detailed in this article illustrates the capability for high fidelity functionally graded prints. Its dual feed, single-output mixing progressive cavity pump extruder allows the user to accurately dose material with precision, and achieve consistent results while printing. This open-source DIW printer is capable of printing a wide range of materials with differing rheological properties, and accurately mixing two components together while extruding. Mixing ratio changes are achievable on-the-fly, and can easily be varied throughout a print through minimal post-processing. The hardware has also been proven to be highly modular and adaptable to print a wide range of materials. Furthermore, the majority of the major components are open source in nature, leaving the user to implement their own modifications to suit their specific printing needs. Using components that are available to the general public, the printer is accessible and relatively inexpensive compared to commercially available mixing extruder DIW printers.

## CRediT authorship contribution statement

**Max J. Sevcik:** Writing – review & editing, Writing – original draft, Visualization, Software, Resources, Methodology, Investigation, Formal analysis, Data curation, Conceptualization. **Jacob Golson:** Visualization, Software, Methodology, Investigation, Formal analysis, Conceptualization. **Gabriel Bjerke:** Resources, Methodology, Investigation, Formal analysis, Conceptualization. **Isaac Snyder:** Formal analysis, Data curation. **Gage Taylor:** Formal analysis, Data curation. **Finnegan Wilson:** Writing – review & editing, Writing – original draft, Resources, Methodology, Investigation, Formal analysis, Data curation, Conceptualization. **Grace I. Rabinowitz:** Writing – review & editing, Writing – original draft, Investigation, Formal analysis, Data curation. **Dylan J. Kline:** Writing – review & editing, Writing – original draft, Supervision, Resources, Project administration, Methodology, Conceptualization. **Michael D. Grapes:** Resources, Methodology. **Kyle T. Sullivan:** Supervision, Project administration, Funding acquisition, Conceptualization. **Jonathan L. Belof:** Supervision, Project administration, Funding acquisition. **Veronica Eliasson:** Writing – review & editing, Writing – original draft, Supervision, Resources, Project administration, Funding acquisition, Conceptualization.

## Declaration of competing interest

The authors declare that they have no known competing financial interests or personal relationships that could have appeared to influence the work reported in this paper.
